# Computational Design and Characterisation of Gyroid Structures with Different Gradient Functions for Porosity Adjustment

**DOI:** 10.3390/ma15103730

**Published:** 2022-05-23

**Authors:** Leonie Wallat, Patrick Altschuh, Martin Reder, Britta Nestler, Frank Poehler

**Affiliations:** 1 Institute of Materials and Processes, Karlsruhe University of Applied Sciences, Molkestr. 30, 76133 Karlsruhe, Germany; 2 Institute for Digital Materials Research, Karlsruhe University of Applied Sciences, Molkestr. 30, 76133 Karlsruhe, Germany; patrick.altschuh@h-ka.de (P.A.); martin_dominik.reder@h-ka.de (M.R.); britta.nestler@kit.edu (B.N.); 3 Institute for Applied Materials-Microstructure Modelling and Simulation, Karlsruhe Institute of Technology, Kaiserstraße 12, 76131 Karlsruhe, Germany

**Keywords:** TPMS structures, sheet-based gyroid, mechanical simulation, modelling, Pace3D

## Abstract

Triply periodic minimal surface (TPMS) structures have a very good lightweight potential, due to their surface-to-volume ratio, and thus are contents of various applications and research areas, such as tissue engineering, crash structures, or heat exchangers. While TPMS structures with a uniform porosity or a linear gradient have been considered in the literature, this paper focuses on the investigation of the mechanical properties of gyroid structures with non-linear porosity gradients. For the realisation of the different porosity gradients, an algorithm is introduced that allows the porosity to be adjusted by definable functions. A parametric study is performed on the resulting gyroid structures by performing mechanical simulations in the linear deformation regime. The transformation into dimensionless parameters enables material-independent statements, which is possible due to linearity. Thus, the effective elastic behaviour depends only on the structure geometry. As a result, by introducing non-linear gradient functions and varying the density of the structure over the entire volume, specific strengths can be generated in certain areas of interest. A computational design of porosity enables an accelerated application-specific structure development in the field of engineering.

## 1. Introduction

Triply periodic minimal surfaces (TPMS) are three-dimensional cell structures that occur in nature in many forms: for example, in butterfly wings [1] or on the skeletal plate of a sea urchin [2]. There are a variety of different structures: for example, gyroid, Schwarz diamond, and Schwarz primitive structures [3], which are defined by a mathematical periodic function and whose surfaces have a mean curvature of zero. This results in a smoothly curved surface, while the periodic cells are divided into two disjointed continuous channels that are intertwined. In addition to their lightweight potential, these cell structures are characterised by unique properties and shapes that make them attractive for a wide range of engineering applications. For example, the high surface-to-volume ratio and the two-phase system are very preferable properties for the development of heat exchangers [4,5,6]. In particular, the work by Weihong Li et al. [5] has shown that a comparison between a printed circuit heat exchanger (PCHE) and a heat exchanger with TPMS structures (Schwarz diamond and gyroid) shows both a higher thermal performance and a higher Nusselt number [5]. Furthermore, TPMS structures are of great interest in the field of tissue engineering, as their topological structure is similar to that of trabecular bone [7]. The introduction of a porosity gradient on the TPMS structures opens up new engineering possibilities. In Dawei Li’s research [8], for example, it was shown that sheet-based linear graded gyroid structures have a high energy absorption potential, which is a crucial property in applications with regard to crash safety. Moreover, the introduction of the gradient offers a new freedom of design. As such, the work of [9] aims to use linear graded cell gradients to replicate the natural environment of bones. Since structures with gradients have so far mostly been investigated with linear gradients and are interesting for a variety of applications, it is desirable to put more emphasis on non-linear porosity adjustments.

**Figure 1:**
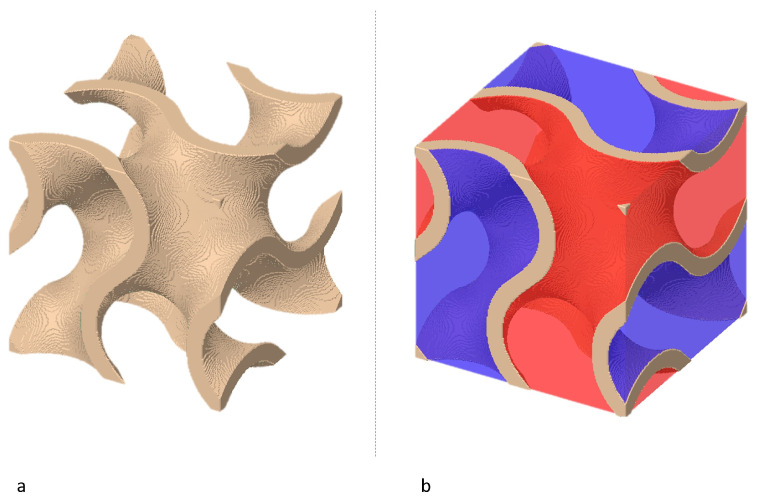
(**a**) Sheet-based gyroid structure; (**b**) gyroid structure with labelled two-tunnel system.

In the following, the group of double gyroid structures is considered, which is exemplified in Figure 1, with the characteristic two-tunnel system. For example, in [8,10], this structure is referred to as ’sheet-based gyroid’ (in the remainder of this article, it will only be referred to as ’gyroid’).

The following equation is used to approximate the surface of the gyroid structure by trigonometrical functions [4].

(1) $$0={\left[\sin\frac{2\pi x}{L_{x}}\;\cdot \;\cos\frac{2\pi y}{L_{y}}+\sin\frac{2\pi y}{L_{y}}\;\cdot \;\cos\frac{2\pi z}{L_{z}}+\sin\frac{2\pi z}{L_{z}}\;\cdot \;\cos\frac{2\pi x}{L_{x}}\right]}^{2}-t^{2}$$

The number of cell repetitions in the *x*-, *y*-, and *z*-directions and the absolute sizes of the unit cells $L_{x}$, $L_{y}$, and $L_{z}$ define the cell space [11]. The thickness of the cell wall is controlled by the variable *t*. Thus, *t* has an effect on the volume fraction ($v^{*}$) of the lattice structure [12]. According to [13,14], the volume fraction ($v^{*}$) and the closely related parameter porosity Φ are defined as follows:

(2) $$\Phi =\left(1-v^{*}\right)\;\cdot \;100\left[\%\right]$$

with

(3) $$v^{*}=\frac{v}{v_{s}},$$

where *v* and $v_{s}$ denote the volume of the pore structure and the volume of the solid structure, respectively [13,14]. In the literature, $v^{*}$ is also referred to as ’relative density’ [14,15]. As can be seen from equation (2), the higher the porosity, the thinner the cell walls. According to Gibson and Ashby [16], the mechanical properties of porous structures of the same topology are directly influenced by their porosity. They propose a correlation between the effective Young modulus and the relative density, which is known as Gibson–Ashby correlation. In addition to the porosity, the mechanical properties are also strongly influenced by the topology of the structure [11,17]. With respect to gyroid structures with imposed porosity gradients, the question therefore arises as to how different geometries with non-linear porosity gradients influence the resulting effective behaviour of the structures. Since the complex manufacture of gyroid structures is cost and time intensive, it is desirable to answer these questions through digital modelling and simulations.

In this work, an algorithm for generating gyroid structures with imposed porosity gradients is introduced, and the resulting mechanical properties of the gyroids are investigated by performing a simulation study. For the structure generation, a constant porosity and two different functions are considered: a linear and a quadratic function. The numerical simulations are performed in the linear elastic regime, which is a common approach in the field of open cell foams. Kaoua et al. [18] use finite element (FE) simulations on Kelvin unit cells to investigate different ligament cross section geometries. In the work by Gan et al. [19] and Zhu et al. [17], elastic FE simulations are also applied to Voronoi-based foams, whereby in the latter work, the influence of geometry irregularities is investigated. The aim of the work is to enable the digital design of gyroid structures with tailored porosity gradients for specific applications.

## 2. Computational Design

**Figure 2:**
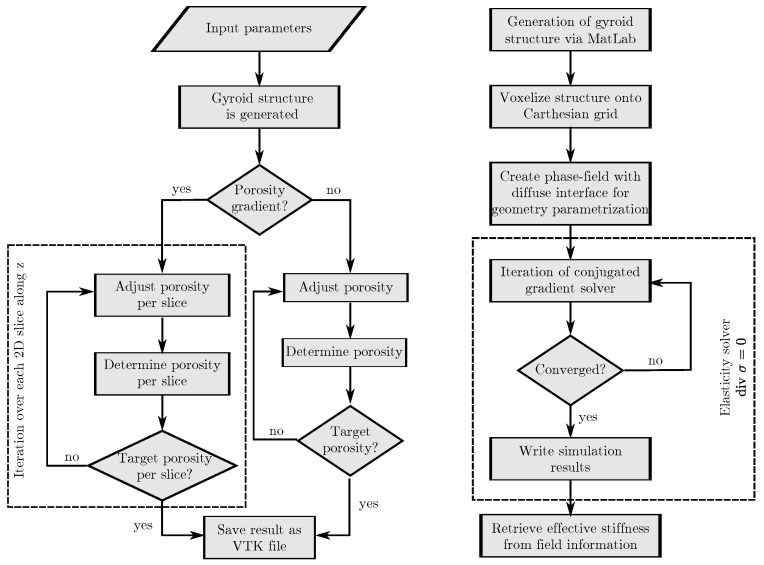
Schematic representation of the computational design of the structures. Generation of the gyroid structures in the MatLab program (**left**) and the workflow for the mechanical simulations with Pace3D (**right**).

Before mechanical simulations of the structures can be performed, the digital structures are created on the basis of a spatial algorithm. For the structure creation, a MatLab [20] source was programmed, which enables the creation of TPMS structures with and without gradients. The aim of the MatLab program is to create gyroid structures with adjustable porosities and definable porosity gradients, using mathematical functions. The TPMS structures are stored in vtk files, while the further preprocessing of the structures as well as the simulations are realised with the simulation framework Pace3D [21]. The simulation software “Parallel Algorithms for Crystal Evolution in 3D” (Pace3D) is a massive parallel in-house software package [21] which is developed at the Institute for Digital Materials Science (IDM) of the Karlsruhe University of Applied Sciences, Germany. The objective of Pace3D is to provide a package for large-scale multiphysics simulations, so as to solve coupled problems such as solidification, grain growth, mass and heat transport, fluid flow and mechanical forces (elasticity, plasticity), etc. The use of dimensionless quantities enables a scale-independent representation of the results, so that the simulations are performed with a non-dimensionalisation. With the help of a conversion table, physical quantities can be obtained from the results. The corresponding flowchart from the creation of the structures in the MatLab program to the mechanical simulation is summarised in Figure 2.

### 2.1. Structure Generation

#### 2.1.1. Input Parameters

The MatLab program offers the possibility of creating structures with constant and graded porosity. Table 1 lists and briefly describes the required input parameters for the structure generation, while the individual parameters and their influence are discussed more specifically in the following.

The input parameters $num_{x}$, $num_{y}$, and $num_{z}$ specify the number of unit cells to be repeated in the corresponding direction, while the parameter unitCellSize defines the physical dimension of the created gyroid unit cell. By multiplying unitCellSize with the input parameters $num_{x}$, $num_{y}$, and $num_{z}$, the quantities $L_{x}$, $L_{y}$, and $L_{z}$ of Equation (1) are obtained, which also represent the total domain size of the resulting structure in physical dimensions. The parameter $n_{\mathrm{steps}}$ defines the number of voxels that are used to discretise the gyroid in all directions of one spatial unit cell.

With $\Phi_{\min}$ and $\Phi_{\max}$, the range of the minimum and maximum porosity is defined. In the MatLab program, the porosity is specified in the range between [0; 1], which represents the more commonly used expression of 0% and 100% for the porosity. When creating a gyroid structure with a constant overall porosity, the value from $\Phi_{\max}$ is used. The porosity function is defined with the parameter func. The polynomial degree of the gradient function can be selected between 0 and 2, corresponding to a constant (0), a linear (1), and a quadratic (2) representation. The input parameter grad determines whether the gyroid structure is generated with or without gradient by the integer values 1 and 0, respectively. Per default, the gradient occurs in the *z*-direction.

The parameter delta is mainly responsible for the iterative adjustment of the actual porosity to the target porosity. A tolerance range is defined that describes the maximum permissible deviation between the actual porosity of the current layer and the target porosity. For instance, if the parameter is set to a value of 0.02, this corresponds to a deviation of the actual data of 2%, compared to the target porosity function. The smaller the number of this parameter, the more accurate and longer the program takes to calculate. The target porosity per cell layer is calculated by the gradient function.

**Figure 3:**
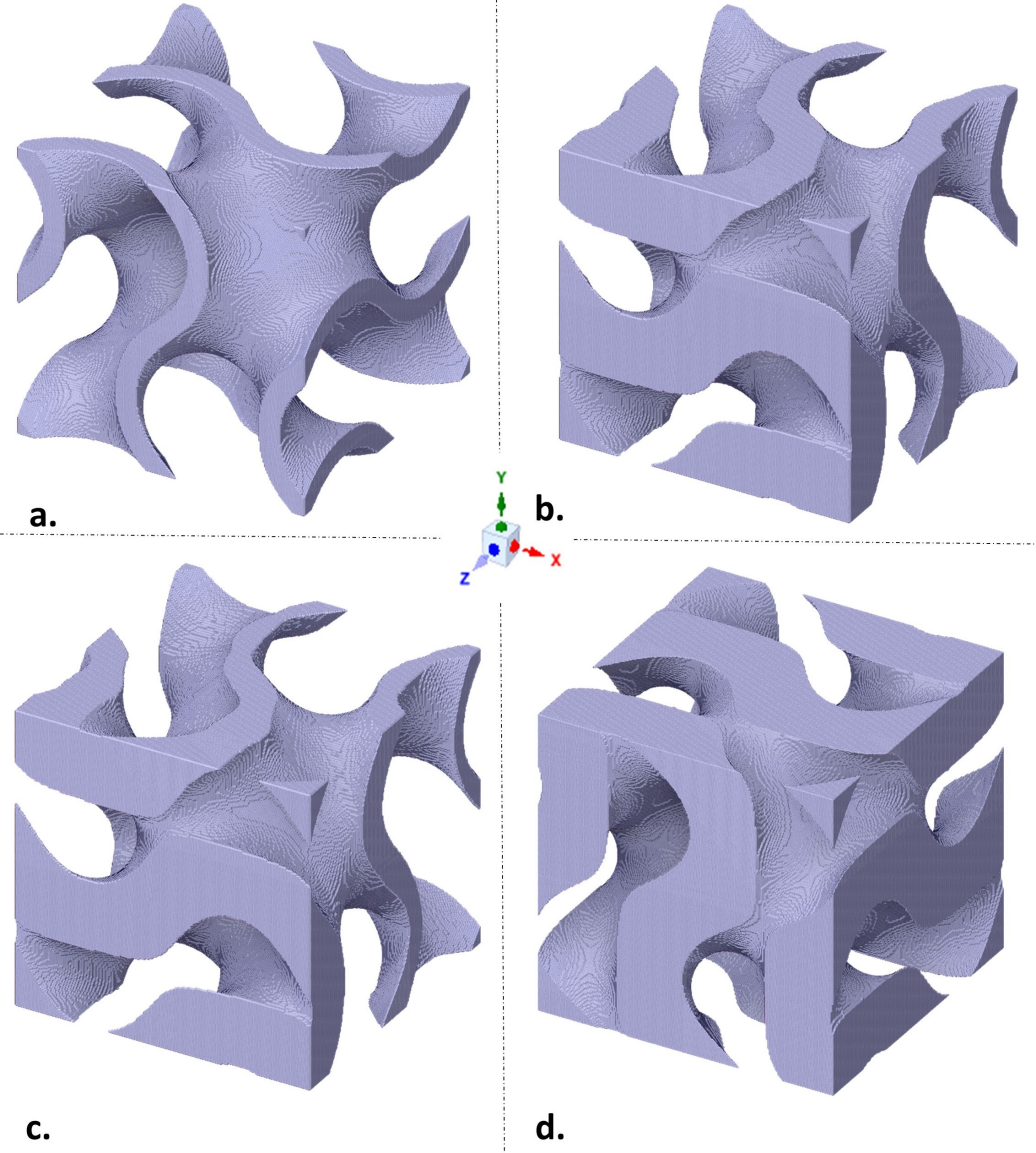
Unit cell of the gyroid structure; gradient with (**a**) constant function, with a porosity of 0.8; (**b**) linear function, with a porosity from 0.4 to 0.8; (**c**) quadratic function, with a porosity from 0.4 to 0.8; (**d**) constant function, with a porosity of 0.4.

Figure 3 shows four different gyroid unit cell structures with a cell size of 2.5 mm, which is generated with 200 steps and a delta parameter of 0.02. Figure 3a,d illustrate a gyroid unit cell with a constant porosity of 0.8 (a) and 0.4 (d). By looking at the two structures, the influence of the porosity on the cell thickness becomes evident. The higher the porosity, the thinner the wall thickness. The structures Figure 3b,c refer to different gradient functions: linear (b) and quadratic (c) functions. The structures are in a porosity interval between 0.4 and 0.8.

#### 2.1.2. Algorithm

In the algorithm, the porosity is adjusted step by step. First, the total volume of an initially generated cell structure is calculated as a starting point, while the discrete voxel values in the domain vary between −1 and 1, according to Equation (1). Once the gradient function is selected, the superimposition begins in an iterative process. Three encapsulated for-loops are used to iterate over the spatial domain and a while-loop is responsible for adjusting the porosity to the target porosity. The adjustment is made by applying a threshold with values between −1 and 1, which divides the domain into structure space and tunnels. The specified tolerance limits are used as the termination criterion of the while loop. In this way, the target porosity for each 2D layer of the 3D structure can be adjusted according to the gradient function. If no gradient is selected, the porosity adjustment is not applied per layer but to the entire cell structure. For the definition of the gradient function ($\Phi_{\mathrm{target}}$), a choice between the following three functions is possible so far.

Constant function:

(4) $$\Phi_{\mathrm{target}}=\Phi_{\max}$$

Linear function:

(5) $$\left.\Phi_{\mathrm{target}}=-\frac{\Phi_{\max}-\Phi_{\min}}{n_{{\mathrm{steps}}_{z}}}\;\cdot \;q+\Phi_{\max}\right.$$

Quadratic function:

(6) $$\Phi_{\mathrm{target}}=-\frac{\Phi_{\max}-\Phi_{\min}}{{\left(n_{{\mathrm{steps}}_{z}}-3\right)}^{2}}\;\cdot \;{\left(q-2\right)}^{2}+\Phi_{\max}$$

The constant function calculates a structure with a constant porosity along each spatial direction. For the linear cell gradient, a linear function with the usual linear structure y=a*x+b is used. The first part of the equation calculates the stepwise increase in the cell volume in each cell level (or the decrease in the porosity). Here, the calculation depends on the number of discrete points ($n_{\mathrm{steps}}$) in whose direction the gradient is imposed. In this case, the gradient extends into the *z*-direction. The second part of the formula is used to determine the initial porosity $\Phi_{\max}$. In the first layer, the structure has the porosity of $\Phi_{\max}$, which is gradually decreased until the final porosity $\Phi_{\min}$ is reached. *q* is the index parameter for the outermost for-loop and at the same time the spatial position of the current 2D layer.

**Figure 4:**
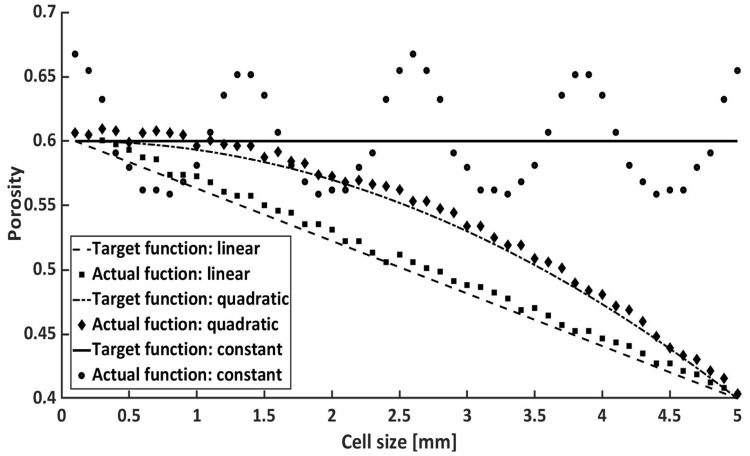
Porosity function of the constant, linear, and quadratic function.

For the quadratic cell gradient, a quadratic function of the structure $y=a*x^{2}+b*x+c$ is used with (b=0). As with the linear function, a stepwise decrease in the porosity (increase in the cell volume) is determined at each cell level, except that the decrease should be quadratic. Since the quadratic gradient is defined as an inverted parabola with an offset on the y-axis, at the level of maximum porosity, the gyroid structure with a quadratic gradient is thickened more slowly than those with a thickening linear structure, as can be seen in Figure 4.

In Figure 4, the three different gradients (constant, linear, and quadratic) are compared with their target and actual values of a single-cell gyroid structure. In each case, 50 actual and 50 target values per porosity function (input parameter: $n_{\mathrm{steps}}=50$) are mapped over ’Cell size’ [ 5 mm], in the *z*-direction [*x*-axis], and ’Porosity’ [*y*-axis]. The maximum porosity is 0.6, and the minimum porosity is 0.4. The actual values are an approximation of the target values. As already mentioned, the fit of the objective function mainly depends on the delta parameter.

### 2.2. Model and Setup for Mechanical Simulations

For the mechanical simulations, the static momentum balance in the small deformation regime is solved with a finite element discretisation. This is done using the Pace3D framework, which employs a phase-field method for the geometry parametrization. Therefore, the structures generated in the MatLab program (see Section 2.1) are discretised on a Cartesian grid, and a diffuse interface is employed between the metal and the surrounding air.

From the micromechanics-microstrcture simulations, the stress tensor σ and the strain tensor ϵ are obtained as full field information. This gives rise to the normalised von Mises stresses $\sigma_{\mathrm{VM}}$, whose maximum value determines the start of local plastification if it reaches the yield strength of the materials. Through homogenisation, an effective Young modulus can additionally be obtained from the stress and strain field [22]. This is done using the volume-averaged stress and strain over the whole computational domain and relating them via the effective Young modulus.

Simulations of compression tests are performed with the specified stress $\sigma_{\mathrm{BC}}$, which is applied in the *z*-direction, as the boundary condition on both sides of the simulation domain. All other boundaries are set to be stress-free. The domain is discretised using a Cartesian grid with 200×200×200 elements. The air phase between the structure is modelled with a stiffness of zero, while the solid phase is considered to exhibit an isotropic elastic material behaviour.

## 3. Results and Discussion

### 3.1. Structure Consideration

Table 2 lists all 13 gyroid unit cells created for the subsequent investigation by mechanical simulations. Overall, the structures differ only in their porosity and gradient function. As can be seen from Table 2, the structures range in porosity from 0.4 to 0.8. For the structures with graded porosity (linear and quadratic), the thickening of the structures always ends at a porosity of 0.4. It should be mentioned that when the volume decreases, the influence on the mechanical stability should be considered, so as to optimise the lightweight potential. Since the porosity has a significant influence on the surface-to-volume ratio, and thus on the mechanical stability, it is also taken into account. The other input parameters that do not change are listed in Table 3. Assuming that the gyroid structures are characterised by the periodicity of their unit cell in all directions, it should be possible to apply the results of the mechanical simulation to multicell structures. In all three directions (*x*, *y*, *z*), the size of the analysed cell is set to 2.5 mm. A unit cell is divided into 200 voxels per spatial direction ($n_{\mathrm{steps}}$ = 200). The possible deviation from the target function is 2% (delta=0.02).

### 3.2. Surface Area-to-Volume Ratio

The surface area-to-volume (SA/V) ratio is an important technical aspect. A high SA/V ratio, for example, favours more efficient heat exchange [23] but usually has negative effects on the mechanical properties, which is why the SA/V ratio of the gyroid cells is investigated. To calculate the surface area-to-volume ratio, the stl files created in the MatLab program were imported into the Ansys workbench [24], where the volume and surface area of each structure were output.

**Figure 5:**
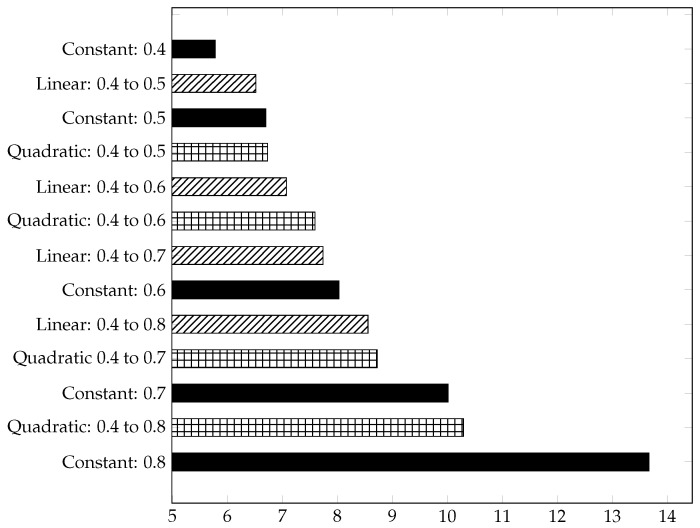
Surface [mm${}^{2}$]/volume [mm${}^{3}$] of gyroid structures in ascending order.

The bar chart Figure 5 lists the SA/V ratio from the gyroid structures in ascending order, with and without gradients. There, it can be seen that a high porosity favours the ratio. For this reason, the gyroid cell with a constant porosity of 0.8 has the highest SA/V ratio of all structures. In contrast, the gyroid with a porosity of 0.4 has the lowest ratio. The structures with a quadratic gradient have a higher SA/V ratio than those with a linear gradient. This is due to the fact that the structure with a quadratic gradient thickens more slowly, as can be seen in Figure 4. Between the gyroid structures with a constant porosity of 0.5 and 0.6, there are four structures with gradients. In the course of this work, a possible correlation between the SA/V ratio and the mechanical properties will be considered. 

### 3.3. Mechanical Simulation

Material-independent and relative statements depending on the porosity type can be made about the structures with the same load scenario. For this reason, scaled data are used. For the analysis of the structures, a steady-state case with an applied load of $\sigma_{\mathrm{BC}}=400\;MPa$ is considered. Since the simulations are performed within the linear-elastic regime, the results are independent of the structure material and the characteristic length, due to the linear scalability. The effective Young modulo as well as the maximum and mean values of the von Mises stress are evaluated. The latter values correspond respectively to the volume average ${\bar{\sigma }}_{\mathrm{VM}}$ of the von Mises equivalent stress field and its maximum value $\sigma_{\mathrm{VM},\max}$ within the domain. Note that both quantities are given normalised with the load $\sigma_{\mathrm{BC}}$ and can thus be interpreted as mean and maximum values of a stress amplification factor. Dividing the yield strength of the material under consideration (e.g., AlMg7Si0.6) by this amplification factor gives the actual limit for local plastification and thus an effective yield strength. The effective Young modulus is given normalised with the one of the structure materials. In order to obtain physical quantities, a multiplication can be carried out with the material value under consideration. For example, ${\bar{E}}_{AlMg7Si0.6}=E_{AlMg7Si0.6}\bar{E}$, with $E_{AlMg7Si0.6}=59\;GPa$, if the structure is made of the alloy AlMg7Si0.6. The use of these normalised quantities allows a comparison between the structures without specifying the material or length scale.

**Figure 6:**
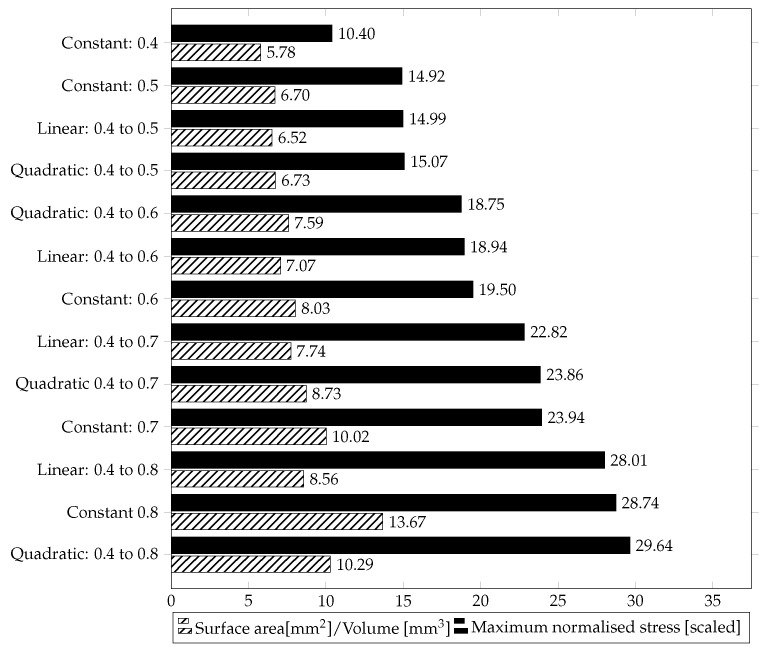
Dimensionless, scaled maximum normalised stress $\sigma_{\mathrm{VM},\max}$ [scaled] of gyroid structures in ascending order, in comparison to the SA/V ratio of the same structure.

**Figure 7:**
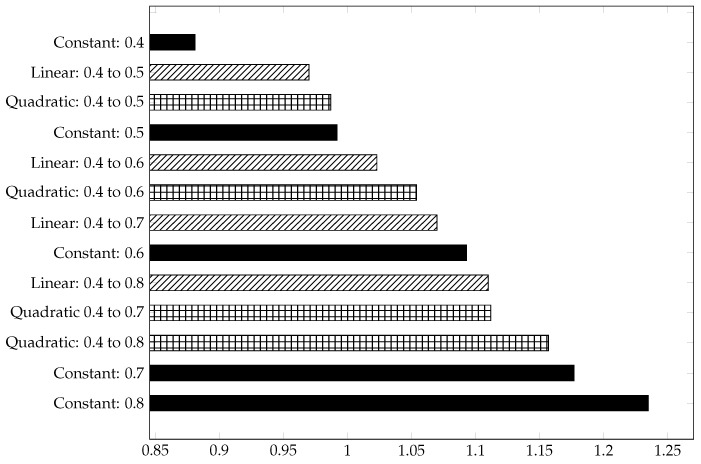
Dimensionless, scaled mean normalised stress ${\bar{\sigma }}_{\mathrm{VM}}$ of gyroid structures in ascending order.

**Figure 8:**
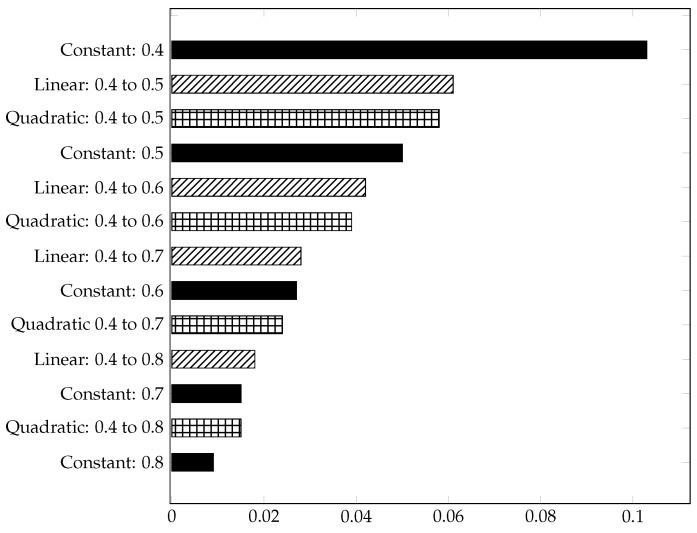
Dimensionless, scaled effective Young modules $\bar{E}$ of gyroid structures in decreasing order.

Table 4, Table 5 and Table 6 represent the material-independent and scaled values of the respective structures. For better clarity and comprehensibility of the results, they are also shown in the bar charts Figure 6, Figure 7 and Figure 8. The structures in the charts are all labelled according to the following pattern: ’Type of gradient function: Porosity interval’. ’Quadratic: 0.4 to 0.8’, for example, means that a gyroid structure with a quadratic gradient function and a porosity interval between 0.4 and 0.8 is considered.

In Figure 6, the black bars represent the maximum normalised stresses ($\sigma_{\mathrm{VM},\max}$) of the gyroid structures, sorted in ascending order. In addition, the corresponding SA/V ratio is shown in striped bars.

The gyroid structures with a quadratic porosity have the highest (quadratic: 0.4 to 0.8) and the gyroid structure with constant porosity has the lowest (constant: 0.4) maximum stresses, respectively. In addition, the bar graph illustrates that the linear gradient structures have lower scaled normalized maximum stresses than the quadratic gradient structures, but higher than the constant gradient structures. A structure with a linear porosity of 0.4 to 0.6 corresponds to an average of a constant porosity of 0.5. Here, it becomes clear that by adjusting the gradient, a higher SA/V ratio is achieved, but also higher maximum stresses. This observation also applies to the structure with a linear porosity from 0.4 to 0.8, which corresponds to an average porosity of 0.6.

As in Figure 6, the dimensionless, scaled, and normalised stresses [${\bar{\sigma }}_{\mathrm{VM}}$] of the structures are also sorted in ascending order in Figure 7. This allows for a faster comparison of the diagrams. The structural arrangements of the two diagrams are not in the same order, since there is a deviation between the highest and the lowest values of the maximum normalised stress and the mean normalised stress.

Compared to the structures with quadratic or linear porosity, the structures with constant porosity have higher normalised stresses. The higher the porosity level of the structures, the higher the normalised stresses. This may indicate a more uniform stress distribution in the structures with gradients or that the structure has more unstressed regions. The gyroid structure ’quadratic: 0.4 to 0.8’, for example, has a higher SA/V ratio than ’constant: 0.7’, but a lower mean normalised stress.

The structures with a linear gradient generally exhibit the lowest mean stresses, compared to the other structures, which is also reflected in the scaled, dimensionless elastic modulus (see Figure 8).

The structures with a linear gradient have a higher effective Young modulus [$\bar{E}$] than the other structures in the same porosity range. This can be partly explained by the fact that the linear gradient structures are thickened more quickly and more evenly, which means that the initiating force can be better distributed. The Figure 8 lists the scaled and dimensionless effective Young modulus of the considered structures in decreasing order. It becomes clear that the porosity has a high influence on the effective Young modulus. Between the structures ’constant: 0.4’ and ’constant: 0.8’, for example, the effective Young modulus is reduced by a factor of about 11. In contrast, the effective Young modulus for the structures ’quadratic: 0.4 to 0.5’ (’linear: 0.4 to 0.5’) and ’quadratic: 0.4 to 0.8’ (’linear: 0.4 to 0.8’) decreases by a factor of 3.9 (quadratic) or 3.4 (linear). A clear relationship between the SV/A ratio and the effective Young modulus can be seen when comparing Figure 5 and Figure 8. The sorted effective Young modulus is almost in the same order as the sorted SV/A ratio.

**Figure 9:**
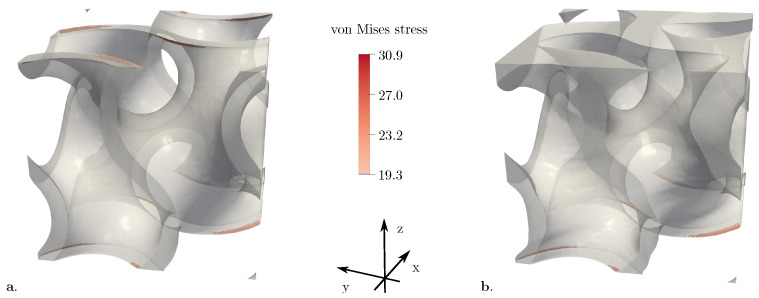
Maximum stresses on the surface of the gyroid structure with (**a**) a constant porosity of 0.8; (**b**) a quadratic porosity function between 0.4 and 0.8.

The evaluation of the von Mises stress field on the gyroid structures revealed that it is generally located at the rounded edges of the structure. It was noticed that the stress peaks for the gyroid structures with gradients are on the side with the highest porosity, due to the difference in porosity, while the stress peaks for the structures with constant porosity are on both sides, which can be seen in Figure 9. The stressed areas are marked in red and are located at the rounded edges, as described previously. Accordingly, the loaded edges are likely to fail first in compression tests. In addition, the one-sided loading of the structures with gradients would explain the higher stress distributions given in Figure 6.

The mechanical simulation has shown that the structures with gradients enable new design and lightweight construction possibilities. The structures with gradients can be better adapted to the required properties. Depending on the choice of the new and the original structure, one property can be specifically improved, while another property can be reduced. In general, it can be said that the SA/V ratio increases with increasing porosity, while the effective Young modulus decreases. By choosing the structure ’linear: 0.4 to 0.5’, for example, instead of ’constant: 0.5’, the effective Young modulus increases by about 22% [quadratic gradient 16%], while the surface-to-SA/V ratio and the mean normalised stress would decrease by about 2% [quadratic about 0%].

If only the mechanical stability is of interest and not the lightweight potential or the surface area-to-volume ratio, the gyroid with the lowest porosity—in our case 0.4—is still unbeatable.

Likewise, the gyroid with a constant porosity of 0.8 would be interesting for applications where only the surface-to-volume ratio is of central importance, but not the mechanical properties.

## 4. Conclusions

In this study, gyroid structures, which are associated with the TPMS family, were investigated. In addition to the used structures with constant porosity, graded structures were produced. For the graded structures, a distinction was made between structures with linear and quadratic gradients. For the mechanical simulation, the created structures were imported into Pace3D. The results were converted into dimensionless, material-independent indices, so that a general statement could be made. In addition to the mechanical simulation, the SV/A ratio was also analysed.

The mechanical simulation shows that the introduction of the gradient multiplies the range of engineering design possibilities. Depending on the desired property and application, it is worthwhile to integrate a gradient into the structure. In general, it can be said that the structures with gradients usually have higher stress peaks, but lower mean normalised stresses.

For future applications, it would be interesting to create more gradient functions. One possibility, for example, would be to create a structure with a gradient which is thickened hourglass-like in terms of volume fraction, since the stress peaks all occur in the outer edge region. In addition, an experimental validation of the results would also be important, which is part of the ongoing work. The combination of experimental data and dimensionless indices will allow a tailor-made design for individual parts in the future.

## Figures and Tables

**Table 1:** Input parameters of the MatLab program and their function for the creation of a gyroid structure.

Input Parameter	Function
$num_{x}$	
$num_{y}$	Number of unit cells to be repeated in the *x*-, *y*-, and *z*-direction
$num_{z}$	
unitCellSize	Size of the unit cells (in mm)
$n_{\mathrm{steps}}$	Resolution of the unit cell
$\Phi_{\max}$	Maximum and minimum porosity of the cell
$\Phi_{\min}$	
func	Gradient function
grad	With/without gradient function (1, 0)
delta	Tolerance range

**Table 2:** Created gyroid structures: constant gradient, linear gradient, quadratic gradient.

Constant Gradient	Linear Gradient	Quadratic Gradient
0.4	-	-
0.5	0.4 to 0.5	0.4 to 0.5
0.6	0.4 to 0.6	0.4 to 0.6
0.7	0.4 to 0.7	0.4 to 0.7
0.8	0.4 to 0.8	0.4 to 0.8

**Table 3:** Non-varying input parameters across all structures.

Input Parameter	Value
$num_{x}$	1
$num_{y}$	1
$num_{z}$	1
unitCellSize [mm]	2.5
$n_{\mathrm{steps}}$	200
delta	0.02

**Table 4:** Scaled results of the gyroid structure with a constant gradient [dimensionless] of the normalised effective Young modules $\bar{E}$, a mean von Mises stress ${\bar{\sigma }}_{\mathrm{VM}}$, and a maximum von Mises stress $\sigma_{\mathrm{VM},\max}$, which is given for different porosities.

Porosity	$\bar{E}$	${\bar{\sigma }}_{\mathrm{VM}}$	$\sigma_{\mathrm{VM},\max}$
0.4	0.10	0.88	10.40
0.5	0.05	0.99	14.92
0.6	0.03	1.09	19.50
0.7	0.02	1.18	23.94
0.8	0.01	1.24	28.74

**Table 5:** Scaled results of the gyroid structure with a linear gradient [dimensionless] of the normalised effective Young modules $\bar{E}$, a mean von Mises stress ${\bar{\sigma }}_{\mathrm{VM}}$, and a maximum von Mises stress $\sigma_{\mathrm{VM},\max}$, which is given for different porosities.

Porosity	$\bar{E}$	${\bar{\sigma }}_{\mathrm{VM}}$	$\sigma_{\mathrm{VM},\max}$
from 0.4 to			
0.5	0.06	0.97	14.99
0.6	0.04	1.02	18.94
0.7	0.03	1.07	22.82
0.8	0.02	1.11	28.01

**Table 6:** Scaled results of the gyroid structure with a quadratic gradient [dimensionless] of the normalised effective Young modules $\bar{E}$, a mean von Mises stress ${\bar{\sigma }}_{\mathrm{VM}}$, and a maximum von Mises stress $\sigma_{\mathrm{VM},\max}$, which is given for different porosities.

Porosity	$\bar{E}$	${\bar{\sigma }}_{\mathrm{VM}}$	$\sigma_{\mathrm{VM},\max}$
from 0.4 to			
0.5	0.06	0.99	15.07
0.6	0.04	1.05	18.75
0.7	0.02	1.11	23.86
0.8	0.02	1.16	29.64

## Data Availability

Not applicable.

## References

[1] Michielsen K., Stavenga D. (2008). Gyroid cuticular structures in butterfly wing scales: Biological photonic crystals. J. R. Soc. Interface.

[2] Lai M., Kulak A.N., Law D., Zhang Z., Meldrum F.C., Riley D.J. (2007). Profiting from nature: Macroporous copper with superior mechanical properties. Chem. Commun..

[3] Kladovasilakis N., Tsongas K., Tzetzis D. (2021). Mechanical and FEA-Assisted Characterization of Fused Filament Fabricated Triply Periodic Minimal Surface Structures. J. Compos. Sci..

[4] Alketan O., Abu Al-Rub R. (2019). Multifunctional mechanical-metamaterials based on triply periodic minimal surface lattices: A review. Adv. Eng. Mater..

[5] Li W., Yu G., Yu Z. (2020). Bioinspired heat exchangers based on triply periodic minimal surfaces for supercritical CO_2_ cycles. Appl. Therm. Eng..

[6] Torquato S., Donev A. (2004). Minimal surfaces and multifunctionality. Proc. R. Soc. A Math. Phys. Eng. Sci..

[7] Dong Z., Zhao X. (2021). Application of TPMS structure in bone regeneration. Eng. Regen..

[8] Li D., Liao W., Dai N., Xie Y.M. (2019). Comparison of Mechanical Properties and Energy Absorption of Sheet-Based and Strut-Based Gyroid Cellular Structures with Graded Densities. Materials.

[9] Liu F., Mao Z., Zhang P., Zhang D.Z., Jiang J., Ma Z. (2018). Functionally graded porous scaffolds in multiple patterns: New design method, physical and mechanical properties. Mater. Des..

[10] Jin Y., Kong H., Zhou X., Li G., Du J. (2020). Design and Characterization of Sheet-Based Gyroid Porous Structures with Bioinspired Functional Gradients. Materials.

[11] Maskery I., Sturm L., Aremu A., Panesar A., Williams C., Tuck C., Wildman R., Ashcroft I., Hague R. (2018). Insights into the mechanical properties of several triply periodic minimal surface lattice structures made by polymer additive manufacturing. Polymer.

[12] Maskery I., Aboulkhair N., Aremu A., Tuck C., Ashcroft I. (2017). Compressive failure modes and energy absorption in additively manufactured double gyroid lattices. Addit. Manuf..

[13] Chen Z., Xie Y., Wu X., Wang Z., Li Q., Zhou S. (2019). On hybrid cellular materials based on triply periodic minimal surfaces with extreme mechanical properties. Mater. Des..

[14] Feng J., Liu B., Lin Z., Fu J. (2021). Isotropic porous structure design methods based on triply periodic minimal surfaces. Mater. Des..

[15] Zaharin H., Abdul-Rani A.M., Azam F., Ginta T., Sallih N., Ahmad A., Yunus N.A., Zulkifli T.Z.A. (2018). Effect of Unit Cell Type and Pore Size on Porosity and Mechanical Behavior of Additively Manufactured Ti6Al4V Scaffolds. Materials.

[16] Gibson L.J., Ashby M.F. (1997). Cellular Solids: Structure and Properties.

[17] Zhu H., Hobdell J., Windle A. (2000). Effects of cell irregularity on the elastic properties of open-cell foams. Acta Mater..

[18] Kaoua S.A., Boutaleb S., Dahmoun D., Azzaz M. (2016). Numerical modelling of open-cell metal foam with Kelvin cell. Comput. Appl. Math..

[19] Gan Y., Chen C., Shen Y. (2005). Three-dimensional modeling of the mechanical property of linearly elastic open cell foams. Int. J. Solids Struct..

[20] MATLAB (2019). Version 9.6.0.1072779 (R2019a).

[21] Hötzer J., Reiter A., Hierl H., Steinmetz P., Selzer M., Nestler B. (2018). The parallel multi-physics phase-field framework Pace3D. J. Comput. Sci..

[22] John A., John M. (2016). Foam metal and honeycomb structures in numerical simulation. Ann. Fac. Eng. Hunedoara.

[23] Planinsic G., Vollmer M. (2008). The surface-to-volume ratio in thermal physics: From cheese cube physics to animal metabolism. Eur. J. Phys..

[24] Ansys (2021). Version 2021 R2.

